# Environmental and socio-economic determinants of infant mortality in Poland: an ecological study

**DOI:** 10.1186/s12940-015-0048-1

**Published:** 2015-07-21

**Authors:** Agnieszka Genowska, Jacek Jamiołkowski, Krystyna Szafraniec, Urszula Stepaniak, Andrzej Szpak, Andrzej Pająk

**Affiliations:** Department of Public Health, Faculty of Health Sciences, Medical University of Bialystok, Bialystok, Poland; Department of Epidemiology and Population Studies, Institute of Public Health, Faculty of Health Sciences, Jagiellonian University Medical College, Krakow, Poland

**Keywords:** Birth outcomes, Working conditions, Industrial environment, Health inequalities, Ecological study, Poland

## Abstract

**Background:**

Health status of infants is related to the general state of health of women of child-bearing age; however, women's occupational environment and socio-economic conditions also seem to play an important role. The aim of the present ecological study was to assess the relationship between occupational environment, industrial pollution, socio-economic status and infant mortality in Poland.

**Methods:**

Data on infant mortality and environmental and socio-economic characteristics for the 66 sub-regions of Poland for the years 2005–2011 were used in the analysis. Factor analysis was used to extract the most important factors explaining total variance among the 23 studied exposures. Generalized Estimating Equations model was used to evaluate the link between infant mortality and the studied extracted factors.

**Results:**

Marked variation for infant mortality and the characteristics of industrialization was observed among the 66 sub-regions of Poland. Four extracted factors: “poor working environment”, “urbanization and employment in the service sector”, “industrial pollution”, “economic wealth” accounted for 77.3 % of cumulative variance between the studied exposures. In the multivariate regression analysis, an increase in factor “poor working environment” of 1 SD was related to an increase in infant mortality of 40 (95 % CI: 28–53) per 100,000 live births. Additionally, an increase in factor “industrial pollution” of 1 SD was associated with an increase in infant mortality of 16 (95 % CI: 2–30) per 100,000 live births. The factors “urbanization and employment in the service sector” and “economic wealth” were not significantly related to infant mortality.

**Conclusion:**

The study findings suggested that, at the population level, infant mortality was associated with an industrial environment. Strategies to improve working conditions and reduce industrial pollution might contribute to a reduction in infant mortality in Poland.

**Electronic supplementary material:**

The online version of this article (doi:10.1186/s12940-015-0048-1) contains supplementary material, which is available to authorized users.

## Background

Infant mortality, an important indicator of population health [1], is higher in Poland than is the average in EU-15 countries (4.73 vs. 3.59 per 1000 live births) [2] and varies considerably between the provinces of the country [3]. The geographical differences in infant mortality rates could be partially explained by the differences in environmental exposures, including environmental factors in the workplace, and outdoor and indoor pollution [4–6]. The disparities in infant health could also appear as an effect of the differences in socio-economic, behavioral, and medical factors and access to health services [7–9].

In Poland, perinatal conditions associated with pregnancy disorders (mainly shortening the duration of pregnancy and fetal disorders) contribute to 52 % of infant mortality and over 30 % of infant mortality could be attributed to birth defects [10].

The dynamic industrialization in Poland in the second half of the 20th century resulted in high levels of environmental pollution, particularly air and water pollution [11]. The quality of drinking water is worse compared to the average for Organization for Economic Co-Operation and Development countries (OECD) [12]. Moreover, compared with EU countries, a higher percentage of employees in Poland feel threatened by working conditions, e.g., chemicals, dusts, fumes, smoke or gases, as well as noise and vibration [13]. There is evidence that environmental pollution and poor working conditions are related to low birth weight [14–16], preterm birth [17, 18], birth defects [19–21] and infant mortality [5, 22].

Numerous toxic substances can act during the preconceptional period and affect the reproductive system in both sexes [6, 23]. Particularly dangerous are lipophilic chemicals (pesticides, polychlorinated biphenyls, dioxins) which are stored in body fat and may be mobilized during pregnancy and lactation. These chemicals may pass through the placental barrier and affect the fetus [24]. Fetuses and infants are susceptible to chemical substances more than adults, due to their fast growth, the immaturity of their defense mechanisms and their weight [25, 26]. During pregnancy, a placenta may accumulate toxic metals (e.g., lead), which may result in reduced blood flow, deterioration of the transport nutrients and fetal growth restriction [27].

On the other hand, infant mortality is closely related to socio-economic factors, e.g., educational level, gross domestic product per capita, and income inequality [7]. There is evidence that the level of education of pregnant women determines their health behaviors, such as diet, smoking or alcohol consumption [4, 28, 29].

Associations between environmental hazards and birth outcomes are not consistent across other studies, the results of which have a number of limitations to their interpretation [5]. Studies on infant mortality are particularly important in countries where infant mortality is high. Poland is one such country where environmental determinants of the health status of infants are poorly recognized [30]. The aim of the present ecological study was to assess the relationship between occupational environment, industrial pollution, socio-economic status and infant mortality in Poland.

## Methods

### Research design and data source

An ecological design was applied to study the relationships between infant mortality and environmental and socio-economic determinants at an aggregate level. We used data from the 66 sub-regions of Poland, defined as NUTS-3 (Nomenclature of Units for Territorial Statistics), according to the recommendations of the European Union [31]. Information on infant mortality rates, and indices of urbanization, occupation, occupational conditions, environmental pollution and socio-economic status in the years 2005–2011 were obtained from the Central Statistical Office of Poland. A detailed list of the original predictors is presented in Additional file 1: Table S1.

### Data analysis

A total of 23 independent variables measured at the sub-region level were used in the analysis. As most variables had a statistically significant deviation from the normal distribution, they were transformed before analysis. For dealing with skewness and simultaneously accounting for unique properties of variables that include a zero value, the inverse hyperbolic sine transformation [32] was used according to the formula:

$$ f\left(x,\theta \right)=\frac{sin{h}^{-1}\left(\theta x\right)}{\theta } $$

The *θ* parameter was chosen, so that the distribution of transformed variables was as similar as possible to the normal distribution.

As correlation analysis revealed strong correlations within some groups of independent variables, to avoid the multicollinearity problem in regression modeling, factor analysis was used. Factor analysis places together closely related items to form a smaller number of “latent variables” (factors) explaining the maximum amount of common variance in a correlation matrix. Each factor consists of all items, but the importance of individual variables corresponds to factor loadings. Therefore, individual factors are scores that represent different dimensions of source data. To identify factors, Principal Component Analysis with orthogonal rotation (equamax) on factor loadings was used [33]. This allowed a reduction of the large number of variables to a smaller number of mutually uncorrelated factors [34]. Finally, four factors were extracted that explained 77.3 % of the cumulative variance of the original dataset. Factors were given descriptive names based on their intercorrelation structure. For this purpose, variables with absolute factor loadings above 0.6 were considered noteworthy (Additional file 2: Table S2). This criterion allowed for unequivocal naming, as none of the variables were highly correlated with more than one factor (the variable “cold microclimate” was not substantially correlated with any factor). The factors were assigned the following names: “poor working environment” (explained 23.2 % of variance), “urbanization and employment in the service sector” (explained 20.8 % of variance), “industrial pollution” (explained 19.7 % of variance), “economic wealth” (explained 13.6 % of variance). As extracted factors were composed of a set of variables measured in different units, the factors themselves have no particular unit; rather, they are standardized scores (with mean = 0 and standard deviation (SD) = 1). Factors should be interpreted as hidden characteristics underlying the observed variables used to create them and the factor loadings describe which variables (and in which direction) are represented the most by a given factor [35].

To assess the relationship between infant mortality and the four extracted factors, the Spearman correlation was calculated. This simple analysis was then extended with a more complex multivariate regression model with the infant mortality ratio as the dependent variable and all four factors as independent ones, taking into consideration the presence of the correlated data (time-repeated measurements of 7 years of observation). We employed the Generalized Estimating Equations modeling approach [36] assuming an exchangeable structure for the working correlation matrix. The results are presented as the expected change of infant mortality rate per 100,000 live births for an increase in a given factor of one unit which was equal to 1 SD.

To describe the average annual change of the infant mortality rate during the studied period (2005–2011), a simple regression model was used in each sub-region separately and the outcome was presented graphically on a map. Statistical analyses were conducted with IBM® SPSS® Statistics for Windows, Version 20.0-IBM Corp. Armonk, NY, USA.

## Results

**Fig. 1:**
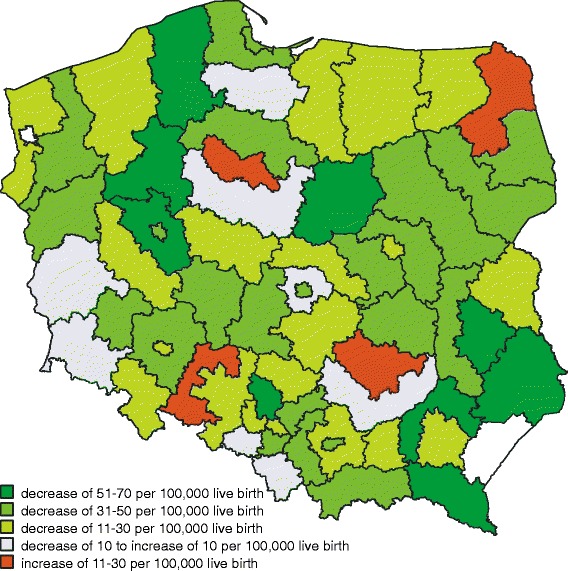
Average annual change in infant mortality rate between 2005 and 2011 by sub-region

In Poland, there were 15,458 infant deaths from 2005 to 2011. In the sub-regions studied, the mean infant mortality rate for the period 2005–2011 ranged from 402 to 766 per 100,000 live births (Table 1). Observed temporal changes in the infant mortality varied by sub-region. However, in the majority of sub-regions, the infant mortality rate decreased during the study period (Fig. 1). The largest annual decrease was one of 69 per 100,000 live births. The highest increase of infant mortality rate was of 26 per 100,000 live births per year.

**Table 1:** Descriptive statistics for 66 Polish sub-regions (average for 2005–2011)

	$\overline{\mathrm{x}}$ (SD)	Me	Q1–Q3	Range
Infant mortality [n/10^5^ live births]	568 (122)	565	517–616	402–766
Urbanization				
Urban population [%]	59 (20.4)	54	46**–**70	23**–**100
Employment structure				
Employment in industry and construction [%]	30 (8.9)	31	24**–**37	14**–**57
Employment in trade, repair, transportation and gastronomy [%]	16 (5.6)	15	13**–**18	7**–**33
Employment in finance and real estate [%]	3 (1.8)	3	2**–**3	1**–**12
Employment in other services [%]	27 (6.2)	26	22**–**31	16**–**42
Employment in agriculture [%]	24 (15.8)	22	12**–**32	1**–**59
Hazards in the work environment				
Strenuous working conditions [n/10^4^ of working]	181 (143.6)	135	80**–**246	14**–**686
Chemical substances [n/10^4^ of working]	24 (34.9)	15	9**–**26	2**–**267
Fibrosis, including industrial dusts [n/10^4^ of working]	55 (128.5)	9	5**–**28	0**–**739
Noise [n/10^4^ of working]	224 (133.6)	215	120**–**277	41**–**767
Vibrations [n/10^4^ of working]	19 (23.9)	13	8**–**22	2**–**183
Hot microclimates [n/10^4^ of working]	20 (39.3)	9	4**–**18	1**–**291
Cold microclimates [n/10^4^ of working]	18 (18.4)	13	4**–**21	2**–**112
Mechanical factors [n/10^4^ of working]	88 (55.6)	72	48**–**110	21**–**256
Industrial pollution				
Total particle pollution [tonnes/km^2^]	1 (1.4)	0	0**–**1	0**–**9
Sulfur dioxide [tonnes/km^2^]	6 (10.9)	1	0**–**4	0**–**46
Nitrogen oxides [tonnes/km^2^]	3 (5.4)	1	0**–**2	0**–**23
Industrial waste [tonnes/km^2^]	1,200 (2,813.6)	129	59**–**757	11**–**16,507
Untreated wastewater [dam^3^/km^2^]	1 (4.9)	0	0	0**–**39
Socio-economic situation				
Gross enrollment rate in tertiary education level [%]	4 (5.9)	2	1**–**5	0**–**24
Industrial production sold [PLN per inhabitant]	20,439 (12,976)	16,081	11,996**–**23,711	4,178**–**67,317
Average salary [PLN per inhabitant]	2,757 (387)	2,655	2,482**–**2,860	2,328**–**4,320
Gross domestic product [PLN per inhabitant]	30,214 (12,871)	26,148	22,777**–**31,945	18,377**–**98,691

$\overline{\mathrm{x}}$ mean value; *SD* standard deviation; *Q1* 1st quartile; *Me* median; *Q3* 3rd quartile

Descriptive statistics for the 66 Polish sub-regions (average values for 2005–2011) are presented in Table 1. There was a large variation in socio-economic characteristics by sub-region, including education, industrial production sold, average salary and gross domestic product. A large variation in the percentage of urban population and employment structure was observed between sub-regions. On average, 30 % of people were employed in industry and construction, 46 % in services and 24 % in agriculture. Among the hazards of the work environment, noise and strenuous work conditions were the most frequent. Also, the differences between sub-regions in terms of industrial pollution were particularly large.

**Fig. 2:**
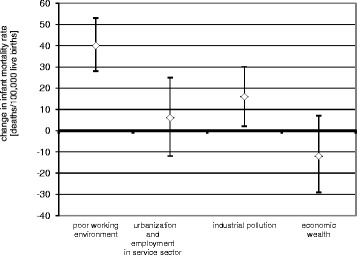
Expected change in the infant mortality per an increase of 1 SD of extracted factors

A positive correlation was found between mean infant mortality rate (average for the period 2005–2011) and the extracted factor “poor working environment” (*r* = 0.58, *P* < 0.001), while the relationships between infant mortality and the extracted factors “urbanization and employment in the service sector” (*r* = 0.12, *P* = 0.345), “industrial pollution” (*r* = 0.21, *P* = 0.097) and “economic wealth” (*r* = 0.06, *P* = 0.651) were not statistically significant. However, in the multivariate regression analysis, factors “poor working environment” and “industrial pollution” were both statistically significant independent determinants of the infant mortality rate. An increase in factor “poor working environment” of 1 SD was related to an increase in the expected infant mortality rate of 40 (95 % CI: 28–53) per 100,000 live births, *P* < 0.001 (Fig. 2). A similar relationship was found for “industrial pollution” factor, where an increase of 1 SD was associated with an increase in the expected infant mortality rate of 16 (95 % CI: 2–30) per 100,000 live births. The relationships between the infant mortality rate and the extracted factors “urbanization and employment in the service sector” and “economic wealth” were not statistically significant with changes of 6 (95 % CI: −12–25) and −11 (95 % CI: −29–7) per 100,000 live births per 1 SD change in these factors).

## Discussion

Infant mortality varied widely in 66 sub-regions of Poland. Results of factor analysis demonstrated that this variation may partially be explained by two factors extracted from 23 characteristics of the 66 sub-regions, i.e. “poor working environment” and “industrial pollution”. Two other extracted factors, i.e. “urbanization and employment in the service sector” and “economic wealth”, were not associated significantly with the infant mortality rate. An interpretation of model parameters should consider the fact that all factors have standardized distributions, which implies that a 0 value for a factor corresponds to the mean value for the population of the sub-regions in Poland and a one-unit change of a factor value corresponds to its change by 1 SD.

### Strengths

As far as we are aware, this is the first study to measure the combined effect of working environment and industrial pollution on infant mortality in Poland. Using the unique analysis of complex relationships between environmental factors and infant mortality, we were able to achieve a high statistical power after including data from 7 years of observation in the 66 sub-regions. Factor analysis enabled the approximate determination of hidden phenomena that manifest themselves in different ways via many individual indicators. This multivariate correlation method is well-suited for revealing underlying patterns or structure among variables showing high degrees of inter-correlation, as in the case of environmental risk variables supposedly influencing health status [37]. Our approach enabled the identification of four independent environmental characteristics out of 23 variables. Two of them appeared to be significantly related to the infant mortality.

### Limitations

The most important limitation is the ecological study design, which does not allow either firm conclusions to be drawn in terms of relationships on an individual level (ecological fallacy) or on causal relationships. Second, the study allowed for limited control of confounding factors, as the appropriate information was not available from public statistics. For example, in Poland 10 % of pregnant women experienced alcohol consumption at least once during pregnancy. The proportion of alcohol consumers was higher among more educated women and women living in informal relationships [38]. Information on the distribution of alcohol consumption among pregnant women by sub-region was not available, but there is evidence of regional variation in alcohol consumption in Poland. The amount of alcohol consumption during pregnancy varied from 0.06 units to 1.19 units per week. Also, information on smoking prevalence by sub-region was not available; however, there is evidence of regional variation in smoking rates among women in general (26.5–64.9 %) and pregnant women in particular (2.7–17.2 %) [38]. Third, our basic data were collected via the national routine statistics system, which put the methods of data collection and data quality beyond our control. Some information obtained from the Central Statistical Office was not comprehensive. For example, data on occupational hazards included only data on workplaces employing more than nine persons [39]. Fourth, in our analysis we focused on environmental factors, but characteristics of the health status of mothers and their access to health services were not controlled directly. However, the latter factors are strongly correlated with economic wealth and urbanization [7, 40].

Our results could be partially explained by the confounding effect of disparities in access to health services by place of residence (urban/rural) [41]. There is evidence that in some regions high risk pregnancies are not identified early enough in prenatal care and there are problems in referring such cases to specialists. Also, there are issues in terms of the equipment of intensive care units for premature infants [42, 43]. Moreover, there is evidence concerning health and life-style problems in pregnant women, including anemia in about 30 %, hypertension in about 6 %, diabetes in about 5 %, smoking in about 7.8 % and alcohol consumption in about 10 %. Nevertheless, it is worth stressing that the vast majority of pregnant women (98 %) are covered by prenatal care in the first trimester [44]. Also, there are observations on positive changes in the diet of the pregnant women, i.e. an increased intake of fresh fruit and vegetables, as well as dairy products, in over 60 % of women [38].

Furthermore, the relationship between the extracted factors and infant mortality might be modified by the timing of the infant death. Addressing this problem was not possible at the population level because information on neonatal and post-neonatal mortality is not available from routine statistics.

### Plausibility

Causes of health conditions leading to infant death are not fully understood [18]. It is likely that other, potentially modifiable environmental factors could play an important role. Generally, our results are in accordance with results from other populations. Low birth weight, which increases the risk of infant mortality, is related to poor working environment [15, 45]. Moreover, exposure to harmful substances at work (e.g., lead, mercury, arsenic, benzene, phthalates) is associated with birth defects or low birth weight [46–48]. However, poor working conditions do not necessarily imply a direct threat to the development of the fetus in mothers exposed, as this relationship has also been shown with the exposure of fathers [6, 49, 50]. Poor occupational environments may act as a marker of other traits, e.g., socio-economic position, which affects the development of the fetus through lifestyle behaviors, psychosocial stress or access to health care [7, 51]. In another study, work in harmful conditions combined with smoking and drinking was associated with the risk of fetal and neonatal death [22].

In our analysis, infant mortality was related to the extracted factors which combined characteristics of industrial pollution. Harmful effects of exposure to air pollution during pregnancy have been observed in many studies [16–18]. Prenatal exposure to air pollution can influence fetal growth due to oxidative stress and inflammation, which may lead to lower birth weight, impaired lung development, early changes in immune development and higher infant mortality. This risk is often observed in populations with low socio-economic status [52]. Higher health risks associated with living and working in areas of heavy industry, e.g., coal mining, metallurgy, smelting, petrochemical, have been found to be related to the release of heavy metals, sulfur compounds, and polycyclic aromatic hydrocarbons and to the presence of fine particulate matter PM_2.5_ and PM_10_, which worsens the quality of water and air [53, 54]. Relationships between women of reproductive age living near hazardous waste landfills and risks of birth defects, low birth weight, prematurity have been described in many studies [14, 20]. In the EUROHAZCON study in European populations, authors found that living during pregnancy within 3 kilometers of a landfill, as well as water pollution, significantly increased the risk of chromosomal and non-chromosomal birth defects [19].

In our study, the combined effect of “urbanization and employment in the service sector” was not related to infant mortality, which may be related to the impact of employment structure, (i.e. unequal distribution by category of employment according to the division in Table 1). The results of other studies are controversial. Higher infant mortality has been associated with living in urban areas in some studies [55, 56], but in others the opposite effect has been demonstrated [57, 58]. Our results could be explained partially by the increase in the employment of rural residents in urban settings, which has occurred recently in Poland, especially in younger generations [59]. The impact of other, not assessed, confounders, such as health behaviors, is also possible.

The factor “economic wealth” was not associated with infant mortality. This finding is in accordance with the fact that Poland is situated at the beginning of the flat part of the Preston curve [60, 61], which means that further economic growth would have relatively small impact on population health. Kim et al. found that associations between macroeconomic characteristics and infant mortality are ambiguous in developed countries [7]. This suggests that the frequently used measure of economic position (GDP per capita) may not be the best marker of the complex characteristics which affect infant mortality.

### Conclusion

The study findings suggested that infant mortality was associated with industrial environmental conditions at the population level. The combined effect of the population characteristics, which are indicated by factors such as “poor working environment” and “industrial pollution”, was related to higher infant mortality and might contribute to explaining the geographical variation in that mortality within a country. On the other side, the effect of the population characteristics, which are indicated by factors such as “urbanization and employment in the service sector” and “economic wealth”, was small and not statistically significant. Strategies to improve working conditions and reduction of industrial pollution might contribute to a decrease in infant mortality in Poland.

## Additional files

Additional file 1: Table S1. Definitions of variables used. Additional file 2: Table S2. Factor loadings from Principal Component Analysis.

## Abbreviations

EU-15: Countries prior to the expansion in May 2004 (Austria, Belgium, Denmark, Finland, France, Germany, Greece, Ireland, Italy, Luxembourg, Netherlands, Portugal, Spain, Sweden and United Kingdom)
SD: Standard deviation

## References

[1] Reidpath DD, Allotey P (2003). Infant mortality rate as an indicator of population health. J Epidemiol Community Health.

[2] World Health Organization: Health for All Database (HFA-DB). http://data.euro.who.int/hfadb (2014). Accessed July 15, 2015.

[3] Wojtyniak B, Stokwiszewski J, Goryński P, Poznańska A, Wojtyniak B, Goryński P, Moskalewicz B (2012). Długość życia i umieralność ludności Polski. Sytuacja zdrowotna ludności Polski i jej uwarunkowania.

[4] Mattison DR (2010). Environment exposures and development. Curr Opin Pediatr.

[5] Wigle DT, Arbuckle TE, Turner MC, Berube A, Yang A, Liu S (2008). Epidemiologic evidence of relationships between reproductive and child health outcomes and environmental chemical contaminants. J Toxicol Environ Health B Crit Rev.

[6] Burdorf A, Figa-Talamanca I, Jensen TK, Thulstrup AM (2006). Effects of occupational exposure on the reproductive system core evidence and practical implications. Occup Med (Lond).

[7] Kim D, Saada A (2013). The social determinants of infant mortality and birth outcomes in Western developed nations: a cross-country systematic review. Int J Environ Res Public Health.

[8] Smith LK, Budd JL, Field DJ, Draper ES. Socioeconomic inequalities in outcome of pregnancy and neonatal mortality associated with congenital anomalies: population based study. BMJ. 2011; doi:10.1136/bmj.d4306 10.1136/bmj.d4306 PMC3139368 21771825

[9] Tromp M, Eskes M, Reitsma JB, Erwich JJ, Brouwers HA, Rijninks-van Driel GC, et. al. Regional perinatal mortality differences in the Netherlands; care is the question. BMC Public Health. 2009; doi:10.1186/1471-2458-9-102. 10.1186/1471-2458-9-102 PMC2674436 19366460

[10] Główny Urząd Statystyczny: Rocznik Demograficzny 2012. http://stat.gov.pl/cps/rde/xbcr/gus/rs_rocznik_demograficzny_2012.pdf (2012). Accessed July 15, 2015

[11] Cembrzyńska J, Krakowiak E, Brewczyński P (2012). Zanieczyszczenia powietrza pyłem zawieszonym PM_10_ oraz PM_2.5_ w warunkach silnej antropopresji na przykładzie miasta Sosnowiec. Med Środow.

[12] Organization for Economic Co-Operation and Development: Better Life Index. http://www.oecdbetterlifeindex.org/topics/environment (2014). Accessed July 15, 2015..

[13] European Union: Eurostat Database. http://ec.europa.eu/eurostat/data/database (2007). Accessed July 15, 2015.

[14] Elliott P, Briggs D, Morris S, de Hoogh C, Hurt C, Jensen TK (2001). Risk of adverse birth outcomes in populations living near landfill sites. BMJ.

[15] Makowiec-Dąbrowska T, Radwan-Włodarczyk Z, Kozada-Włodarczyk W, Siedlecka J, Wilczyński J (1998). Relationship between chemical exposure in the workplace and the risk of premature delivery, low birth weight and intrauterine growth retardation. Int Arch Occ Env Health.

[16] Morello-Frosch R, Jesdale BM, Sadd JL, Pastor M. Ambient air pollution exposure and full – term birth weight in California. Environ Health*.* 2010; doi:10.1186/1476-069X-9-44. 10.1186/1476-069X-9-44 PMC2919523 20667084

[17] Liu S, Krewski D, Shi Y, Chen Y, Burnett RT (2003). Association between Gaseous Ambient Air Pollutants and Adverse Pregnancy Outcomes in Vancouver, Canada. Environ Health Perspect.

[18] Sagiv SK, Mendola P, Loomis D, Herring AH, Neas LM, Savitz DA (2005). A time series analysis of air pollution and preterm borth in Pensylvania, 1997–2001. Environ Health Perspect.

[19] Vrijheid M, Dolk H, Armstrong B, Abramski L, Bianchi F, Fazarinc I (2002). Chromosomal congenital anomalies and residence near hazardous waste landfill sites. Lancet.

[20] Vrijheid M, Dolk H, Armstrong B, Boschi G, Busby A, Jorgensen T (2002). Hazard potential ranking of hazardous waste landfill sites and risk of congenital anomalies. Occup Environ Med.

[21] Brender JD, Zhan FB, Langois PH, Suarez L, Scheuerle A (2008). Residential proximity to waste sites and industrial facilities and chromosomal anomalies in offspring. Int J Hyg Environ Health.

[22] Gaizauskiene A, Padaiga Z, Mizeriene R (2007). Prediction of perinatal mortality at an stage of pregnancy. Scand J Public Health.

[23] Figà-Talamanca I (2006). Occupational risk factors and reproductive health of women. Occup Med (Lond).

[24] Silbergeld EK, Patrick TE (2005). Environmental exposures, toxicologic mechanism, and adverse pregnancy outcomes. Am J Obstet Gynecol.

[25] Schheuplein R, Charney G, Dourson M (2002). Differential sensitivity of children and adults to chemical toxicity. I. Biological basis.. Regul Toxicol Pharmacol.

[26] Grandjean P, Bellinger D, Bergman A, Cordier S, Davey-Smith G, Eskenazi B (2008). The faroes statement: human health effects of developmental exposure to chemicals in our environment. Basic Clin Pharmacol Toxicol.

[27] Zadorozhnaja TD, Little RE, Miller RK, Mendel NA, Taylor RJ, Presley BJ (2000). Concentrations of arsenic, cadmium, copper, lead, mercury, and zinc in human placentas from two cities in Ukraine. J Toxicol Environ Health A.

[28] Kramer MS (2003). The epidemiology of adverse pregnancy outcomes: an overview. J Nutr.

[29] Gaudineau A (2013). Prevalence, risk factors, maternal and fetal morbidity and mortality of intrauterine growth restriction and small-for-gestational age. J Gynecol Obstet Biol Reprod (Paris).

[30] Zejda J (2010). Środowiskowe zagrożenia stanu zdrowia dzieci – polskie doniesienia epidemiologiczne na tle światowej literatury przedmiotu. Przegl Epidemiol.

[31] European Commission: Regions in the European Union. Nomenclature of territorial units for statistics. NUTS 2006/EU-27. http://ec.europa.eu/eurostat/web/products-manuals-and-guidelines/-/KS-RA-07-020. Accessed July 15, 2015.

[32] Burbidge J, Magee L, Robb A (1988). Alternative Transformations to Handle Extreme Values of the Dependent Variable. J Am Stat Assoc.

[33] Jolliffe I (2002). Principal Component Analysis.

[34] Gorsuch R (1983). Factor Analysis.

[35] Lalloué B, Monnez JM, Padilla C, Kihal W, Le Meur N, Zmirou - Navier D et. al. A statistical procedure to create a neighborhood socioeconomic index for health inequalities analysis. Int J Equity Health. 2013; doi:10.1186/1475-9276-12-21. 10.1186/1475-9276-12-21 PMC3621558 23537275

[36] Zeger SL, Liang KY (1986). Longitudinal data analysis for discrete and continuous outcomes. Biometrics.

[37] Marques RC, Bernardi JV, Dórea JG, Bastos WR, Malm O (2008). Principal component analysis and discrimination of variables associated with pre- and post-natal exposure to mercury. Int J Hyg Environ Health.

[38] Żukiewicz-Sobczak W, Paprzycki P, Zwoliński J (2013). Zachowania zdrowotne kobiet w ciąży.

[39] Główny Urząd Statystyczny: Warunki pracy w 2010 r. http://stat.gov.pl/cps/rde/xbcr/gus/pw_warunki_pracy_2010.zip (2011). Accessed July 15, 2015.

[40] Roberts CL, Algert CS (2000). The urban and rural divide for women giving birth in NSW, 1990–1997. Aust N Z J Public Health.

[41] Główny Urząd Statystyczny: Obszary wiejskie. Powszechny Spis Rolny. http://stat.gov.pl/cps/rde/xbcr/gus/RL_obszary_wiejskie_w_polsce_PSR2010.pdf (2013). Accessed July 15, 2015.

[42] Chazan B, Szymborski J (2012). Poprawa stanu zdrowia matek oraz dzieci przed urodzeniem i noworodków. Zdrowie publiczne i polityka ludnościowa.

[43] Gadzinowski J, Niemiec T (2007). Rola trójstopniowej opieki perinatalnej w opiece medycznej nad noworodkiem w Polsce. Zdrowie kobiet w wieku prokreacyjnym 15–49 lat. Polska 2006.

[44] Euro-Peristat: European Perinatal Health Report. The health and care of pregnant women and babies in Europe in 2010. http://www.europeristat.com/images/doc/Peristat%202013%20V2.pdf (2013). Accessed July 15, 2015.

[45] Croteau A, Marcoux S, Brisson C (2006). Work activity in pregnancy, preventive measures, and the risk of delivering a small-for-gestational-age infant. Am J Public Health.

[46] Castilla EE, Campaña H, Camelo JS (2000). Economic Activity and Congenital Anomalies: An Ecologic Study in Argentina. Environ Health Perspect.

[47] Ahmed P, Jaakkola JJ (2007). Maternal occupation and adverse pregnancy outcomes: a Finnish population-based study. Occup Med.

[48] Al-Saleh I, Shinwari N, Mashhour A, Rabah A (2014). Birth outcome measures and maternal exposure to heavy metals (lead, cadmium and mercury) in Saudi Arabian population. Int J Hyg Environ Health.

[49] Li X, Sundquist J, Kane K, Jin Q, Sundquist K (2010). Parental occupation and preterm births: a nationwide epidemiological study in Sweden. Paediatr Perinat Epidemiol.

[50] Li X, Sundquist J, Sundquist K (2010). Parental occupation and risk of small-for-gestational-age births: a nationwide epidemiological study in Sweden. Hum Reprod.

[51] Biernacka J, Hanke W, Makowiec-Dąbrowska T, Makowska Z, Sobala W (2007). Psychospołeczne uciążliwości środowiska pracy zawodowej kobiet ciężarnych a ryzyko występowania porodu przedwczesnego. Med Pr.

[52] Proietti E, Röösli M, Frey U, Latzin P (2013). Air pollution during pregnancy and neonatal outcome: a review. J Aerosol Med Pulm Drug Deliv.

[53] Biggeri A, Lagazio C, Catelan D, Pirastu R, Casson F, Terracini B (2006). Report on health status of residents in areas with industrial, mining or military sites in Sardinia, Italy. Epidemiol Prev.

[54] Ahern M, Mullett M, Mackay K, Hamilton C (2011). Residence in coal-mining areas and low-birth-weight outcomes. Matern Child Health J.

[55] Guildea ZE, Fone DL, Dunstan FD, Cartlidge PH (2005). Differences in risk of mortality under 1 year of age between rural and urban areas: an ecological study. Public Health.

[56] Hillemeier MM, Weisman CS, Chase GA, Dyer AM (2007). Individual and community predictors of preterm birth and low birth weight along the rural – urban continuum an central Pennsylvania. J Rural Health.

[57] Pampalon R, Martinez J, Hamel D (2006). Does living in rural areas make a difference for health in Québec?. Health Place.

[58] Yi B, Wu L, Liu H, Fang W, Hu Y, Wang Y. Rural–urban differences of neonatal mortality in a poorly developed province of China. BMC Public Health*.* 2011; doi:10.1186/1471-2458-11-477. 10.1186/1471-2458-11-477 PMC3144461 21682907

[59] Rosik P, Stępniak M, Wiśniewski R (2012). Dojazdy do pracy do Warszawy i Białegostoku – alternatywne podejścia metodologiczne. Stud Lok Region.

[60] World Health Organization: Closing the gap in a generation: health equity through action on the social determinants of health. The Final Report of the WHO Commission on Social Determinants of Health. http://www.who.int/social_determinants/thecommission/finalreport/en/ (2008). Accessed July 15, 2015.

[61] European Commission: Eurostat regional yearbook 2013. http://www.trf.sll.se/Global/Dokument/Statistik/externa_rapporter/Eurostat-regional-yearbook-2013.pdf (2013). Accessed July 15, 2015.

