# Supplementary material for: Environmental and socio-economic determinants of infant mortality in Poland: an ecological study
Source: Environ Health. 2015 Jul 21;14:61. doi: 10.1186/s12940-015-0048-1 (PMC4508882; doi:10.1186/s12940-015-0048-1)
Supplement: Additional file 1: Table S1. — Definitions of variables used. [file 12940_2015_48_MOESM1_ESM.docx]

**Supplementary Table I** Definitions of variables used

| **Variable name [unit]** | **Definition** |
| --- | --- |
| Infant mortality  [n/10^5^ live births] | Deaths of children less than 1 year of age |
| **Urbanization** | |
| Urban population [%] | According to the place of residence on 31 December each year |
| **Employment structure** |  |
| Employment in industry and construction [%] | Population employed in mining and quarrying; manufacturing; production and supply of electricity, gas and water; water supply; managing waste water and waste management, remediation; construction |
| Employment in trade, repair, transportation and gastronomy [%] | Population employed in trade, repairs, transport, storage, hotel and catering, information and communications |
| Employment in finance and real estate [%] | Population employed in the insurance and financial sector, real estate market services |
| Employment in other services [%] | Population employed in professional, scientific and technical activities; service activities administration; public administration and national defense, compulsory social security; education; health care and social assistance; activities related to arts, entertainment and recreation; other services |
| Employment in agriculture [%] | Population employed in agriculture, forestry, hunting |
| **Hazards in the work environment** | |
| Strenuous working conditions[n/10^4^ of working] | Threats-cum-persons^a^ related to negative impact on work activities in a forced body position, under conditions of heavy exercise or in terms of the specific nuisance |
| Chemical substances  [n/10^4^ of working] | Threats-cum-persons^a^ related to chemicals (toxic, irritant, sensitizing, carcinogenic or mutagenic) active in the labor process |
| Fibrosis, including industrial dusts [n/10^4^ of working] | Threats-cum-persons^a^ related to dust, which can cause pulmonary fibrosis (dust containing more than 2% of free, crystalline silica or silicates or asbestos) |
| Noise [n/10^4^ of working] | Threats-cum-persons^a^ related to harmful sounds that can cause hearing loss and other changes in the body or materially impede the exercise of work |
| Vibrations [n/10^4^ of working] | Threats-cum-persons^a^ related to vibrations occurring during manual operation of tools or equipment percussion (riveting hammers, pneumatic hammers and chisels, saws, drills, rammers, perforator) and vibration occurring at the work place as a result of the operation of machines and technical equipment |
| Hot microclimates  [n/10^4^ of working] | Threats-cum-persons^a^ related to the work environment defined by the ratio of the thermal load WBGT (Wet Bulb Globe Temperature) |
| Cold microclimates  [n/10^4^ of working] | Threats-cum-persons^a^ related to environment conditions specified by an indicator of the strength of the cooling air WCI (Wind Chill Index) |
| Mechanical factors  [n/10^4^ of working] | Threats-cum-persons^a^ related to mechanical factors associated with particularly dangerous machinery (saws, injection molding machines, milling machines, presses, lifts) |
| **Industrial pollution** | |
| Total particle pollution  [tonnes/km^2^] | Total dust emissions from plants, especially onerous (from fuel combustion, cement - lime, silicon, chemical fertilizers, surfactants-active and polymers, carbon - graphite, carbon black, polymers, coal, chromium, mercury, lead, cadmium, arsenic, zinc, manganese, polycyclic aromatic hydrocarbons) |
| Sulfur dioxide [tonnes/km^2^] | Emissions of sulfur dioxide from the combustion of fuels and processes of plants, particularly onerous |
| Nitrogen oxides [tonnes/km^2^] | Emissions of nitrogen oxides from the combustion of fuels and processes of plants, particularly onerous |
| Industrial waste [tonnes/km^2^] | Waste resulting from industrial activities |
| Untreated industrial waste water [dam^3^/km^2^] | The amount of untreated waste water from production processes and from drainage of mine and buildings, discharged into the ground or water |
| **Socio-economic situation** | |
| Gross enrollment rate in tertiary education level [%] | Number of students in public and private colleges and universities in relation to the population level |
| Industrial production sold  [PLN per inhabitant] | The value of sold production at basic prices (after exclusion of excise tax and value adding subsidies) by entities employing > 9 people |
| Average salary  [PLN per inhabitant] | The ratio of the sum of gross personal monthly salaries, fees paid to certain groups of employees for work under the contract of employment, payments in respect of the share in the profit or balance surplus in co-operatives and additional annual salaries for employees of the public sector to the average number of employees during the period; after the exclusion of out workers and those employed abroad |
| Gross domestic product  [PLN per inhabitant] | The aggregate value of final goods and services produced in Poland during the calendar year, expressed in current prices |

**^a^** Threats-cum-persons consist of the total amount of all hazardous factors influencing a worker. If there is only one hazardous factor per every person the total of threats per capita amounts to the total number of the persons working in hazardous conditions.
