# Supplementary material for: Environmental and socio-economic determinants of infant mortality in Poland: an ecological study
Source: Environ Health. 2015 Jul 21;14:61. doi: 10.1186/s12940-015-0048-1 (PMC4508882; doi:10.1186/s12940-015-0048-1)
Supplement: Additional file 2: Table S2. — Factor loadings from Principal Component Analysis. [file 12940_2015_48_MOESM2_ESM.docx]

**Supplementary Table II** Factor loadings from Principal Component Analysis

| Variable | Factor 1:  poor working environment | Factor 2:  urbanization and employment in service sector | Factor 3:  industrial pollution | Factor 4:  economic wealth |
| --- | --- | --- | --- | --- |
| Urban population | 0.22 | **0.77** | 0.28 | 0.34 |
| Industry and construction | **0.80** | −0.13 | 0.02 | 0.22 |
| Trade, repair, transportation and gastronomy | 0.10 | **0.73** | 0.06 | 0.49 |
| Finance and real estate | 0.09 | **0.84** | 0.20 | 0.31 |
| Other services | 0.07 | **0.90** | 0.20 | 0.18 |
| Agriculture | −0.46 | −**0.66** | −0.24 | −0.44 |
| Strenuous working conditions | **0.69** | 0.43 | 0.35 | 0.17 |
| Chemical substances | **0.67** | 0.24 | 0.13 | 0.12 |
| Fibrosis, including industrial dusts | **0.68** | 0.08 | 0.45 | 0.03 |
| Noise | **0.89** | 0.07 | 0.14 | 0.00 |
| Vibrations | **0.75** | 0.24 | 0.11 | 0.11 |
| Hot microclimates | **0.67** | −0.07 | 0.23 | 0.07 |
| Cold microclimates | 0.33 | 0.29 | −0.32 | −0.16 |
| Mechanical factors | **0.84** | 0.13 | −0.07 | 0.11 |
| Total particle pollution | 0.14 | 0.40 | **0.84** | 0.20 |
| Sulfur dioxide | 0.01 | 0.29 | **0.87** | 0.26 |
| Nitrogen oxides | −0.03 | 0.25 | **0.87** | 0.27 |
| Industrial waste | 0.27 | 0.27 | **0.74** | 0.40 |
| Untreated wastewater | 0.34 | −0.02 | **0.62** | 0.12 |
| Gross enrollment rate in tertiary education level | −0.13 | **0.80** | 0.25 | 0.02 |
| Industrial production sold | 0.29 | −0.01 | 0.25 | **0.82** |
| Average salary | −0.11 | 0.22 | 0.17 | **0.84** |
| Gross domestic product | 0.03 | 0.41 | 0.26 | **0.84** |
| Variance [%] | 23.2 | 20.8 | 19.7 | 13.6 |
| Cumulative variance [%] | 23.2 | 44.0 | 63.7 | 77.3 |

*Absolute factor loadings larger than 0.6 are highlighted
